# Evaluating the feasibility of a web-based weight loss programme for naval service personnel with excess body weight

**DOI:** 10.1186/s40814-017-0122-2

**Published:** 2017-02-06

**Authors:** Gulcan Garip, Kate Morton, Robert Bridger, Lucy Yardley

**Affiliations:** 1 University of Derby Online Learning, Enterprise Centre, Derby, DE1 3LD UK; 2 Academic Unit of Psychology, Faculty of Social and Human Sciences, University of Southampton, Southampton, UK; 3 Environmental Medicine and Sciences, Institute of Naval Medicine, Gosport, UK

**Keywords:** Naval Service, Obesity, Web-based weight loss, RE-AIM framework, Feasibility

## Abstract

**Background:**

Overweight and obesity are a major concern that may influence the operational capacity of the UK Naval Service (NS). This study was conducted to evaluate the feasibility of trialling and implementing a modified web-based weight loss programme for overweight and obese NS personnel.

**Methods:**

The feasibility of a web-based weight loss programme with minimal face to face support was evaluated using a non-randomised design, based on the *Reach, Efficacy, Adoption,* and *Implementation* (RE-AIM) dimensions of a framework designed for analysing implementation of interventions in practice.

**Results:**

It was estimated that 6% (*n* = 58) of eligible NS personnel at recruitment sites were reached, based on personnel’s expressions of interest to take part in the study. The potential efficacy of the intervention was evaluated by analysing participants’ change in weight (kg) in the two groups. Forty-three participants were allocated to the intervention (*n* = 21) or control group (*n* = 22). Website usage was low, with 1.5 sessions accessed on average, over a 12-week follow-up. Changes in body weight over 12 weeks appeared to be observed for participants in the intervention group but not in the control group. The average weight loss observed in the intervention group (mean = −1.9 kg, SD = 2.1) appeared to reach significance, 95% CI [−2.8, −1.0], whereas no significant weight loss was apparent among control group participants (mean = −0.8 kg, SD = 3.8), 95% CI [−2.4, 0.8]. However, this feasibility study was not powered to test for within or group differences. Recruitment rates varied across five NS establishments invited to take part in the study, suggesting that the web-based weight loss programme was not adopted to the same extent across all bases. The online programme was not implemented as intended in terms of regular usage by participants and support provision by physical training instructors.

**Conclusion:**

The results suggest that the intervention may warrant further investigation provided that engagement with the intervention by both staff and participants can be improved.

## Background

Obesity is defined by the World Health Organization as an excess accumulation of fat that may impair one’s health [1]. It is recognized that UK Naval Service (NS) personnel with overweight and obesity are prevalent, and that this is associated with a range of problems for the NS [2, 3]. A survey aimed at identifying the prevalence of obesity in the NS reported that in a random sample of 1596 personnel, stratified by gender and rank, 13% were obese and 42% were overweight based on self-reported body mass index and waist circumference [2]. There may exist a higher risk of being medically downgraded due to obesity-related health problems among NS personnel with excess body weight [4]. NS personnel with excess body weight tend to self-report a diminished capacity for fitness to work [5], which has implications for the operational capability of the NS. Furthermore, obesity is associated with the development of knee and back disorders, which are the most frequently cited reasons for being medically downgraded in the NS [6]. This indicates that overweight and obesity-related health problems result in considerable financial costs for the NS in terms of absenteeism, medical discharges, and rehabilitation [7].

The NS does provide various on-site services to support personnel in managing their weight, including consultations on nutrition with nurses, and opportunities to work with physical training instructors to increase levels of physical activity. Despite these opportunities for face to face support with health professionals, the prevalence of personnel with excess body weight in the NS indicates that overweight and obese personnel may not be engaging with these resources, or that additional, more conveniently accessible resources (e.g. from an individual’s own home) may be necessary which could be provided alongside existing weight management services [2, 8].

There is growing evidence to suggest that web-based weight loss programmes may be efficacious and effective resources of support for overweight and obese people in community samples [9–11] and in the workplace [12–14]. For NS personnel, the workplace may be particularly well-suited for trialling a web-based weight loss programme since personnel spend a substantial amount of their time at NS establishments. This is the first study to evaluate the feasibility of trialling a web-based weight loss programme in the UK military.

### An overview of the POWeR-RN programme

An existing web-based weight loss programme, titled ‘Positive Online Weight Reduction (POWeR)’ [15] was modified for the NS (i.e. POWeR-RN; Royal Navy [RN]) in light of the findings from interviews with 21 overweight and obese NS personnel [8]. The POWeR programme was chosen for modification for the NS context due to the programme’s flexible approach and acceptability among a community sample [15]. Another reason for choosing POWeR was that the programme was designed to be engaging to men, as it had tailored information and examples according to participants’ gender. In addition, the POWeR programme was developed using the free and open-access online LifeGuide software, which allows easy development and modifications of digital behaviour change interventions for different contexts (i.e. the NS) [15]. POWeR-RN was developed to serve as a non-prescriptive, conveniently accessible source of support for overweight and obese NS personnel to manage their weight, as these factors were identified as being particularly important to overweight and obese NS personnel based on the findings from the interview study [8]. Specifically, participants from community samples reported reasons for being unable to adhere to diets such as, feeling deprived as a result of food restrictions, being unable to incorporate necessary changes into their lifestyle, and the effort required to track calories [16, 17]. This non-randomised feasibility study was conducted to evaluate whether the online POWeR-RN programme could be trialled in a randomised controlled trial in the NS to be used in conjunction with existing weight management support provisions.

The existing web-based weight loss programme, POWeR, aims to help users self-manage their weight in the long-term by offering users a flexible approach, rather than strictly prescriptive instructions. The POWeR programme consists of 12 sessions that are designed to be accessed on a once-per-week basis, helping users self-manage their weight by teaching them to use behaviour change techniques that have been identified as key ingredients for successful behavioural weight management, including self-monitoring, and goal setting [17]. A mixed methods approach was used to develop the structure and content of the POWeR programme [16]. A randomised controlled feasibility study of the POWeR programme in primary care was positively received by participants from community samples, with participants in the usual care (*n* = 43) and web-based only (*n* = 45) group having lost, on average, similar amounts of weight (2.30 kg and 2.50, respectively) [18]. The study concluded that delivering the POWeR programme supported by practice nurses in primary care was feasible.

The POWeR programme was modified in five stages prior to being implemented in the NS as the POWeR-RN programme. Stage 1 consisted of a survey study to determine the prevalence of overweight and obese personnel in the NS and assessed potential users’ receptiveness to using a web-based weight loss intervention [2]. Findings from this study indicated a potential role for the POWeR-RN programme based on responses to a question about whether respondents would be interested in using an online weight loss intervention [2]. In stage 2, a systematic review and synthesis of qualitative studies investigating the weight management experiences of overweight and obese people was conducted, resulting in 12 factors that formed a conceptual framework for mapping the POWeR content against the factors overweight and obese people perceived as relevant to weight management [19]. In stage 3, a mapping exercise of the POWeR content against the 12 factors identified in the systematic review was undertaken, confirming that the POWeR programme included all components perceived as relevant by overweight and obese people attempting to manage their weight [20]. Stage 4 consisted of semi-structured interviews with 21 overweight and obese NS personnel, revealing perceived constraints in the NS related to dietary options and making time for physical activity. Lack of knowledge and motivation were also reported by NS personnel as reasons for being unable to maintain a healthy weight. Modifications to the POWeR programme aimed to contextualise and improve the relevance of the examples and scenarios of the original content for a NS sample. The final stage consisted of think aloud interviews with 14 overweight and obese NS personnel who used, on average, 2.5 sessions of POWeR-RN and stated their thoughts about the intervention as they were using it [20].The aim of this study was to investigate how overweight and obese RN personnel interacted with the POWeR-RN intervention and what their perceptions were of the intervention. A session titled ‘Getting more active’ was excluded from POWeR-RN as this session would be insufficient to meet the physical activity requirements for a NS personnel. Face to face support from physical training instructors (PTIs) was incorporated to the feasibility study as most participants from the NS mentioned that the lack of accountability was perceived as a reason for low or no engagement with the POWeR programme [20].

### Research aims

The primary aim of this study was to assess whether it would be feasible to conduct a full-scale randomised controlled trial of the POWeR-RN programme to use among overweight and obese NS personnel to be used alongside provisions for weight management in the NS, UK. The RE-AIM framework comprises five dimensions that can be used in the evaluation of the feasibility of implementation of an intervention (i.e. programme) in practice [21]. This framework assesses an intervention in terms of: *Reach*, *Efficacy*, *Adoption*, *Implementation*, and *Maintenance* [22]. Four of the five dimensions of the RE-AIM framework were used to assess the feasibility of trialling POWeR-RN in the NS using a non-randomised design. The final dimension of maintenance was not assessed in the present study as this was a short 12-week pilot study to evaluate whether it would be feasible to conduct a randomised controlled trial of the POWER-RN programme for overweight and obese personnel in the NS. The maintenance dimension would be more appropriately evaluated in a future randomised controlled trial of the POWER-RN programme. The evaluation of whether it would be feasible to conduct a randomised controlled trial of the POWeR-RN programme was assessed through POWeR-RN’s:

- *reach*, by establishing the proportion of overweight and obese NS personnel who expressed an interest to take part in the study;
- *efficacy*, by establishing whether a difference in weight change over the 12-week period could be observed in those who used the website and a wait-list control group;
- *adoption,* by identifying the number of participants successfully recruited to the POWeR-RN programme, at each establishment invited to participate in the feasibility study;
- and *implementation*, based on the proportion of instances the programme was implemented as intended, i.e. participants used the website at least once a week in their own time or at work for 12 sessions and attended one meeting with a PTI. The feasibility of PTIs’ involvement in recruitment, data collection, and providing minimal face to face support to participants using the website was also assessed as part of evaluating POWeR-RN in terms of implementation.

## Methods

Ethical approval for the study was granted by the Psychology Research Ethics Committee at the University of Southampton and the UK Ministry of Defence Research Ethics Committee (MODREC Reference No: 330/Gen/12). The extension of CONSORT 2010 checklist specifically for pilot trials was used for reporting the study [23].

### Design

This study used a non-randomised design to determine whether it was feasible to trial the online POWeR-RN programme with minimal face to face support from PTIs in the NS. There were two study conditions. Participants in the intervention condition had immediate access to the POWeR-RN programme and minimal face to face support for 12 weeks. Minimal face to face support was designed to consist of one 10-minute meeting at the 2-week point between a participant and a PTI at their respective base to give participants a sense of accountability when using the POWeR-RN programme. Participants in the waitlist-control condition gained access to the POWeR-RN programme, without any face to face support, once follow-up measures had been collected. During the 12 weeks, participants in the waitlist-control group did not receive any support or treatment for weight management. Participants’ group allocation was determined by which NS establishment they were based at. The rationale for having a non-randomised design was to prevent potential contamination occurring due to participants sharing information from POWeR-RN with colleagues and or peers who may be from the same base but in the control group.

### Recruitment

Recruitment commenced in September 2012 and continued until April 2013. A rolling recruitment strategy was employed. From September 2012 to January 2013, PTIs at the five NS bases were responsible for recruiting overweight and obese personnel to the study, informing them about how to access the online questionnaires and the web-based programme, and collecting participants’ baseline height, weight, and waist circumference measurements. The slow flow of participants instigated a change in the original recruitment design from January 2013. Two of the authors (GG and KM) arranged recruitment days via PTIs at each of the bases and set up stalls to recruit eligible personnel to the study. The stalls were set up during pre-organised events (e.g. Health and Wellbeing day), at lunch times near dining areas and in sports centres, where the stall would be visible to large numbers of NS personnel.

### Participants

The target sample size was 60 overweight or obese (based on participants’ body mass index over 25 and waist circumference measurements over 94 cm in men and 89 cm in women), male and female NS personnel, who were fit for service, and were between the ages of 18 and 55 years. It was intended that 30 participants would be recruited for each group.

### Procedure

Five NS shore-based establishments (i.e. bases) were selected and notified to take part in this study by the Director of Naval Physical Development, based on their geographic location, availability of PTIs, and number of NS personnel. All of the NS establishments involved in this study were located in the south of the UK to allow for the researcher to travel to these bases for recruitment, data collection, and briefings. For the purposes of this study, PTIs were tasked to help with recruitment and to provide the minimal face to face support in the intervention group, as such, only bases with PTIs were selected. The selected bases had relatively larger numbers of NS personnel to ensure that the target sample size could be met. As per the study’s non-randomised design, participants from three of the bases were allocated to the intervention group, whilst participants from the remaining two bases were allocated to the control group. Participants were informed at the time of recruitment the group they were allocated to.

Participants were provided with a participant information sheet containing information about the study, including the two conditions, and written informed consent was obtained from all participants. The information sheet to participants requested that participants in the wait-list control group did not engage in weight loss programmes for the duration of the study. Participants in the intervention group were given a link that gave them access to the POWeR-RN website upon completion of the baseline questionnaires. Participants in the control group were given a different link that only gave them access to the baseline questionnaires and were informed that they would have access to the website following completion of their follow-up measures (anthropometric and questionnaire measures) in 12 weeks’ time. Participants were advised to schedule a face to face meeting with a PTI at week 2 of the trial, to discuss their use of the website so far and to provide an opportunity to ask any questions. PTIs were also asked to encourage participants in scheduling these meetings. All participants who had not completed baseline questionnaires received two reminder emails and two telephone calls from the researcher. Participants in the intervention group were required to complete baseline questionnaire measures in order to gain access to the POWeR-RN programme. Participants in the waitlist-control group were informed that they should complete baseline and follow-up questionnaires to gain access to the programme at the end of the study. Follow-up measures were then collected from both the intervention and control groups by PTIs 12 weeks after the participant signed up to the study. There were no financial incentives associated with participation in this study.

### Measures

Participants’ demographic characteristics including gender, age, rank, qualifications, and marital status, were obtained through self-report. The number of participants recruited from each NS base and the number of instances where PTIs were able to deliver minimal face to face support to participants in the intervention group were collected. Height, weight, and waist circumference measurements were obtained by PTIs or the researcher using a stadiometer, weighing scales, and standard tape measure at the study outset and the follow-up point 12 weeks later. Standard operating procedures were followed for anthropometric measurements. Weight was measured in light clothing and without shoes, height measurements were taken without shoes, and waist circumference measurements were taken horizontally halfway between the lowest rib and the top of the iliac crest, roughly in line with the navel [24]. Anthropometric measures were used to calculate body mass index objectively. Participants were also prompted to complete a series of surveys at baseline and 12 weeks later, including a Theory of Planned Behaviour scale [25] developed to access participants’ behavioural, normative, and control beliefs about using the intervention, an adapted version of the treatment self-regulation questionnaire [26] to assess participants’ autonomous, introjected, and extrinsic motivation prior to using the intervention at baseline only, and Godin’s leisure-time exercise questionnaire to measure levels of physical activity [27].

### Data analysis

The RE-AIM framework comprises five dimensions that were used in the evaluation of the feasibility of implementation of an intervention (i.e. programme) in practice [21]. Four of the five dimensions of the framework were used to assess the feasibility of trialling the POWeR-RN programme in the NS in terms of Reach, Efficacy, Adoption, and Implementation [22]. In the context of this feasibility study, the RE-AIM dimensions were operationalised as follows: reach referred to the proportion of the target population that participated in the study, which was calculated by the number of participants who volunteered for the study divided by the estimated number of personnel that would be eligible to take part in the study across the five NS bases multiplied by 100%.

Efficacy was related to the success rate of POWeR-RN based on predefined outcomes for assessing success within the confines of the study, which was explored by calculating the effect size for weight loss between the two conditions. Confidence intervals were calculated for the intervention and control groups separately and were based on calculating the mean difference and standard deviation in weight loss from baseline to follow-up. This study was not powered to test any hypotheses and the purpose of evaluating potential efficacy was to serve as a preliminary check to observe whether participants in the intervention group tended to lose weight compared to participants in the control group.

Adoption referred to the successful recruitment of participants to the study, and the frequencies of the number of participants recruited from each NS base was used. Implementation referred to the extent to which POWeR-RN was implemented as intended (i.e. with minimal face to face support from PTIs) at the selected establishments, and this dimension was evaluated based on the descriptive statistics on the number of sessions used, baseline and follow-up weight measures, drop-out rates, and the extent to which PTIs in the intervention group conducted face to face support sessions. The decision to exclude maintenance from this feasibility study was based on the definition of this dimension, which states that maintenance is the extent to which the programme is sustained over time [21]. Although follow-up data were collected at 12 weeks, this was part of the intended implementation of the intervention and was therefore evaluated within the implementation dimension of the RE-AIM framework.

## Results

Table 1 presents participants’ demographic characteristics broken down by group. There were 21 participants in the intervention group and 22 in the control group. The average age of participants in the intervention group was 34 years (SD = 10.56), compared with 32 years (SD = 9.44) for participants in the control group. Participants’ body mass indices were calculated by weight (in kilograms) divided by height (in metres) squared. Participants’ baseline body mass index ranged from 25.30 to 39.70 (mean = 31.51, SD = 3.33), across both groups. There were no apparent differences in demographics or body mass index between participants in the website versus the waitlist-control group.

**Table 1:** Baseline demographic characteristics

Variable	Website group (*n* = 21)	Waitlist-control group (*n* = 22)
Gender (male)	19 (90%)	20 (91%)
Age (SD)	34 (10.56)	32 (9.44)
Body weight (SD)	95.69 (12.19)	95.92 (10.98)
Qualifications		
GCSE	9 (43%)	15 (68%)
A-levels	4 (19%)	2 (9%)
Diploma	5 (24%)	3 (14%)
Degree	3 (14%)	2 (9%)
Marital status		
Single	6 (29%)	12 (54%)
Married	11 (52%)	7 (32%)
Partner	4 (19%)	3 (14%)
Rank		
Rating	20 (95%)	20 (91%)
Officer	1 (5%)	2 (9%)

Participants were asked to complete baseline questionnaires, and the completion rate was 18 out of 21 in the intervention group, and 4 out of 22 in the wait-list control group. There was an association between the group participants that were allocated to and whether they completed the baseline online questionnaires, where participants in the website group (18 responders; 3 nonresponders) appeared much more likely to complete the online measures compared to those in the waitlist-control group (4 responders; 18 nonresponders). There were no significant differences between participants who responded to the baseline questionnaires and those who did not, based on age, gender, rank, or marital status on an exploratory basis.

### Reach

The target sample size of 60 was not attained after 8 months of recruitment across five NS bases (approximately 3000 NS personnel in total). Recent research indicated that 30% of NS personnel would be categorised as being at an increased risk of developing obesity-related health problems [3], the same eligibility criteria as were applied to the present study. This prevalence rate suggested that almost a third of NS personnel would be eligible to take part in the study, equating to approximately 1000 personnel across the five participating sites, yet of these only 58 expressed interests in taking part in the study. This suggests that the reach of this study could be estimated as approximately 6% across the five bases. It is worth noting that 13 of these participants did not meet the eligibility criteria based on their BMI and waist circumference data, which did not indicate an increased risk for developing obesity-related health problems.

### Efficacy

Based on the follow-up weight data available from 16 participants in the website group, participants lost on average 1.94 kg (SD = 2.12) over 12 weeks, ranging from losing 6.00 kg to 0 kg. Participants in the control group who had their follow-up weight measurements taken (*n* = 10) lost 0.76 kg (SD = 3.80) on average, ranging from losing 6 kg to gaining 5 kg. Changes in body weight over 12 weeks appeared to be observed for participants in the intervention group but not in the control group. The average weight loss observed in the intervention group (mean = −1.9 kg, SD = 2.1) appeared to reach significance, 95% CI [−2.8, −1.0], whereas no significant weight loss was apparent among control group participants (mean = −0.8 kg, SD = 3.8), 95% CI [−2.4, 0.8]. However, this feasibility study was not powered to test for within or group differences. For the purposes of a preliminary check, a small to moderate effect size was found based on participants’ mean change in weight (calculated by subtracting baseline weight from follow-up weight) between the website and control group, Cohen’s *d* = 0.38. This difference was not statistically significant, 95% CI [−0.72, 2.92].

### Adoption

Once the NS establishments were identified and notified, invitations were sent out to PTIs at the selected bases. Two PTIs from each base volunteered to take part in the study. A briefing was held for all PTIs involved in the study. Two of the authors (GG and KM) provided study packs and information about the study and the roles and responsibilities of the PTIs. Initial participant recruitment occurred via PTIs identifying eligible participants, and this generated 15 participants from three of the five NS bases; two bases in the intervention group and one in the control group. At the other two NS bases, the PTIs did not identify any participants for recruitment. In an attempt to achieve the target of 60 participants, the researchers set up stalls on 17 occasions across the five bases to boost recruitment. Eleven of these stalls were set-up at the bases related to the intervention group and 6 stalls were set-up at bases that were allocated to the control condition. Following researchers’ involvement in recruitment, 28 further participants were recruited from four of the bases. It was not possible to recruit any participants from one of the bases despite researcher involvement. The numbers of participants recruited ranged from no participants at one base to 18 participants at the most successful base.

### Implementation

**Fig. 1:**
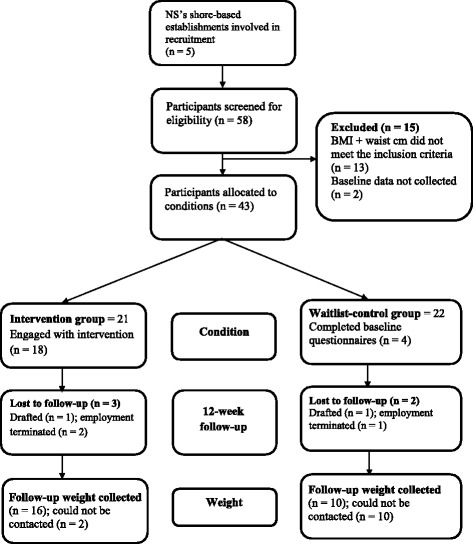
Participant flow through the study

Eighteen participants in the intervention group accessed one or more of the 11 sessions available in the POWeR-RN programme. The average number of sessions participants accessed was 1.5 (SD = 1.29). None of the participants or PTIs arranged the 10-minute face to face supportive meeting which was recommended during the second week of the intervention, and PTIs informed the researcher that this was due to work commitments and not being able to get in touch with participants when PTIs had contacted them. Follow-up weight measurements at 12 weeks were carried out for 26 participants by PTIs at the bases, giving a follow-up response rate of 60%. Attrition was 40% from baseline to follow-up at 12 weeks for the outcome measure of weight (in kg). Figure 1 shows participants’ flow through the study.

## Discussion

The aim of this study was to evaluate the feasibility of trialling and implementing the POWeR-RN programme for overweight and obese personnel in the NS. Based on the evaluations of four (reach, efficacy, adoption, and implementation) out of the five dimensions of the RE-AIM framework, the findings suggest that further development and testing of the POWeR-RN is required prior to trialling the programme in a larger-scale randomised-controlled trial. In terms of the reach of the study, it was not possible to ensure that all eligible personnel were made aware of the study. Clear promotion of the study through the naval hierarchy might help to address this issue. Whilst there was initial support from commanding officers during the recruitment stage of the study, this support subsided once participants had been enrolled to the study, potentially leading to a lack of participant engagement with the study over time. Interviews conducted during the modification process of the POWeR-RN intervention had indicated that support from commanding officers would be required for participants to be able to engage with the intervention during working hours [20]. A number of participants who expressed an interest in participating in the study were ineligible due to their body mass index or waist circumference not exceeding the inclusion criteria threshold. A small proportion of those interested in trialling the website were not classified as overweight/obese, suggesting there was some degree of interest in weight management amongst those who perhaps needed to make just a few, small lifestyle changes to *prevent* their weight becoming a problem.

In terms of efficacy, the findings suggest a weak tendency for participants who used the programme to lose more weight than participants who did not use the programme, however, the study was too small to conclude whether this is a reliable difference. Given that the average website user only logged on to 1.5 out of 11 sessions, it is noteworthy that there was a trend favouring weight loss among participants in the intervention group, though alternative explanations not related to the POWeR-RN programme may be possible. Therefore, the POWeR-RN programme may be efficacious in a definitive trial, especially if steps were taken to improve engagement among participants with the website.

The adoption of the programme by four out of five NS bases is encouraging; however, it is noted that the uptake at some bases was not optimal and it is possible that stronger site-wide adoption and endorsement might have encouraged increased receptiveness to the trial amongst eligible personnel. The implementation of the study suggested that there are barriers to participants’ engagement with the programme, as motivation to engage with the programme appeared to be low. Unless these barriers are addressed, it is unlikely that the programme would be well-received in the future. Based on the relative autonomy index scores collected as an additional part of this study (but not reported in the results for the purposes of the feasibility study), participants appeared to be in the preparation stage of the stages of change model [28], i.e. they were considering losing weight but were not yet ready to start doing it. Consequently, an intervention that incorporates techniques to promote intrinsic motivation to lose weight, such as motivational interviewing, might help them move to the implementation stage. Qualitative process interviews with NS participants might also be useful in the future to provide further insight into participants’ experiences of using online weight management programmes. The lack of face to face support from PTIs suggested that this method of providing additional encouragement to participants in the programme, which was designed to simulate the nurse support provided to civilian populations using POWeR, is not feasible in practice. There is significant evidence for the benefit of face to face encouragement for participants in an online weight management programme [29] and therefore the identification of a more suitable format to provide this support to NS personnel (such as medical staff) would be worthwhile in the future.

The POWeR website was originally designed for middle-aged obese primary care patients, and the content predominantly focused on dietary advice, which may not have been perceived as relevant for managing weight by a relatively physically active sample. Alternatively, it might be beneficial to emphasise the importance of dietary control as a means for weight loss amongst service personnel in healthy lifestyle education sessions, and as a shift in NS culture. Male uptake of weight loss programmes is typically low, with a systematic review of 244 weight loss programmes reporting that male participation was 27% [30]. Web-based weight loss programmes that specifically target younger men with lower levels of education, such as the SHED-IT (Self-Help, Exercise, and Diet using Information Technology) programme, may be better received by the NS population [11]. The SHED-IT programme generated a high interest from over 600 male participants in the community, of which 200 participants were eligible to take part in the study [11]. Another web-based weight loss programme for overweight and obese shift and office workers found that 12% of employees agreed to take part in the study and 5% dropped-out during the trial [31]. However, this difference in uptake may be due to the lack of available resources for weight loss in community samples versus the NS. The overweight and obese NS personnel in our study had greater access to intensive in-person support for weight loss, which may have reduced the motivation to engage with a web-based weight loss intervention for some personnel.

NS personnel undergoing training and living in accommodation on bases may need to pay to use the Internet. In a larger sample, it might be useful to compare website engagement between those with paid internet access and those with free access, or those with office-based positions compared to participants with non-office-based jobs, in order to assess to what extent ease of access to the website affected engagement. It may be useful to know more about when personnel preferred to access the website, as some personnel may not have received support to complete the programme sessions during work time. One of the themes to emerge from an earlier qualitative study highlighted participants’ unwillingness to engage in weight loss behaviours during their own time, but that they would be willing to engage in them during working hours [8]. This may suggest a wider issue in terms of motivation to engage in weight management.

The study was useful for evaluating processes related to the reach, potential efficacy, adoption, and implementation of the programme. As the follow-up period was only 12 weeks, it was not possible to evaluate whether the effects of the programme were maintained in the longer term. However, it appears that engagement with the programme tends to decline after 2 weeks, which suggests that users may not engage with the programme in the long-term without significant changes to the current design and programme implementation. The feasibility study was not designed to evaluate, nor does it make claims about, the effectiveness of the web-based weight loss programme for overweight and obese NS personnel.

### Strengths and limitations

There are several strengths of this study. First, the research was conducted in a group that is currently underrepresented in the weight management literature, i.e. young men with lower levels of education [19, 30, 32]. This study may have higher ecological validity compared to other weight management programmes as there was no introductory information session with participants and no incentives were given for taking part in the study. The existing web-based weight loss programme was evaluated in light of overweight and obese NS personnel’s experiences [8], which is in line with current recommendations for enhancing the acceptability of the programme for target users [19]. The final strength of this study is the application of the RE-AIM framework for evaluating the feasibility of the modified web-based weight loss programme (i.e. POWeR-RN). Based on the current findings, it would be premature to take this programme further in its current form on a larger scale until the identified barriers have been addressed.

There are some limitations of the study. In terms of measures, whilst the same equipment was used to measure participants’ height, weight, and waist circumference at baseline and follow-up where possible, in some cases, different equipment was used which may have led to measurement error. In addition, participants were not specifically asked about their level of motivation due to the assumption that participants signing up to the study would have some level of motivation to lose weight and use the programme. However, the inclusion of these items may have been informative for assessing participants’ level of motivation, given the low levels of engagement that were found. At the end of the study, participants were not asked whether they had engaged in other sources of support for weight loss and this information could have yielded further explanations of the findings. Participants could be sent on deployment at any time which meant that some participants could not be contacted for their follow-up measurements. Finally, this was a descriptive study, and therefore the findings cannot be used to draw any conclusions about the effectiveness or efficacy of the web-based weight loss programme in the NS context at this time.

## Conclusion

This is the first programme of research to evaluate the feasibility of trialling and introducing a modified web-based weight loss programme for overweight and obese personnel in the NS. This study developed our understanding of overweight and obese NS personnel’s (low) engagement with a web-based weight loss programme (i.e. POWeR-RN). Several recommendations for future trials with the web-based weight loss programme have been identified, which, if addressed, could lead to an increase in personnel’s interest and engagement with the online weight loss programme: 1) identifying and introducing sources of support to increase overweight and obese NS personnel’s internal motivation for losing weight, 2) promoting awareness about the importance of diet for losing weight, in addition to engaging in exercise, among overweight and obese NS personnel, 3) identifying a suitable system for providing face to face support to users of the online weight loss programme, and 4) identifying and integrating behaviour change techniques that resonate with overweight and obese NS personnel into the weight loss programme.

Based on the findings from this study, it is concluded that further modifications are required before the online weight loss programme may be trialled and implemented in the NS. Specifically, support is needed to first increase overweight and obese NS personnel’s motivation to self-manage their weight, and qualitative research could inform new ways to increase user engagement with the online weight loss programme.

## Abbreviations

NS: Naval service
POWeR: Positive online weight reduction
POWeR-RN: Positive online weight reduction for the royal navy
PTI: Physical training instructor
RE-AIM: Reach, Efficacy, Adoption, Implementation, Maintenance
SHED-IT: Self-help, exercise, and diet using information technology
UK: United Kingdom
